# Conformational Dynamics of the Receptor-Binding Domain of the SARS-CoV-2 Spike Protein

**DOI:** 10.3390/biomedicines10123233

**Published:** 2022-12-12

**Authors:** Aleksandra A. Mamchur, Tatiana B. Stanishneva-Konovalova, Yuliana A. Mokrushina, Viktoria A. Abrikosova, Yu Guo, Hongkai Zhang, Stanislav S. Terekhov, Ivan V. Smirnov, Igor A. Yaroshevich

**Affiliations:** 1 Department of biophysics, Faculty of Biology, Lomonosov Moscow State University, Moscow 119991, Russia; 2 Department of bioengineering, Faculty of Biology, Lomonosov Moscow State University, Moscow 119991, Russia; 3 Shemyakin-Ovchinnikov Institute of Bioorganic Chemistry, Russian Academy of Sciences, Moscow 117997, Russia; 4 State Key Laboratory of Medicinal Chemical Biology, College of Pharmacy, Nankai University, Tianjin 300071, China

**Keywords:** COVID-19, SARS-CoV-2, receptor-binding domain, molecular dynamics

## Abstract

Variants of SARS-CoV-2 keep emerging and causing new waves of COVID-19 around the world. Effective new approaches in drug development are based on the binding of agents, such as neutralizing monoclonal antibodies to a receptor-binding domain (RBD) of SARS-CoV-2 spike protein. However, mutations in RBD may lower the affinity of previously developed antibodies. Therefore, rapid analysis of new variants and selection of a binding partner with high affinity is of great therapeutic importance. Here, we explore a computational approach based on molecular dynamics simulations and conformational clusterization techniques for the wild-type and omicron variants of RBD. Biochemical experiments support the hypothesis of the presence of several conformational states within the RBD assembly. The development of such an approach will facilitate the selection of neutralization drugs with higher affinity based on the primary structure of the target antigen.

## 1. Introduction

The COVID-19 pandemic is arguably the biggest biomedical challenge of recent years around the world [1]. The causative agent, SARS-CoV-2 virus, is extensively studied across the majority of biological and medical fields, mainly to obtain new effective drugs for fighting the spread and the course of the disease. The first vaccines were developed in less than a year, and therapeutic antibodies soon followed. The applied empirical drug discovery methods performed extremely well and, during that time, got their own development boost [2]. However, the emergence of new variants carries the risk of reducing the effectiveness of previously developed drugs. Moreover, the natural immunity of patients exposed to the previous SARS-CoV-2 variants may not cope with new ones. Therefore, the development of new drugs and new approaches to finding them is of great therapeutic importance. A promising way is the use of a theoretical target-based drug design technique, which requires the knowledge of a target and its structure. Structural methods such as X-ray and cryo-EM may be of great help here, but highly flexible target regions represent a challenge for them. This study focuses on the receptor binding domain (RBD), and more precisely, its receptor binding motif (RBM), of glycoprotein S in the wild type (often labeled as Wuhan-1) and omicron variants of the SARS-CoV-2.

SARS-CoV-2 infects the human body by entering epithelial cells of the respiratory tract. This process starts when glycoprotein S (spike protein, S-protein) binds to the angiotensin-converting enzyme 2 (ACE2) receptor located on the surface of the plasma membrane. As a consequence, the development of critical conditions in the human body can occur [3,4,5,6,7], which makes it extremely important to develop new drugs to fight SARS-CoV-2.

**Figure 1:**
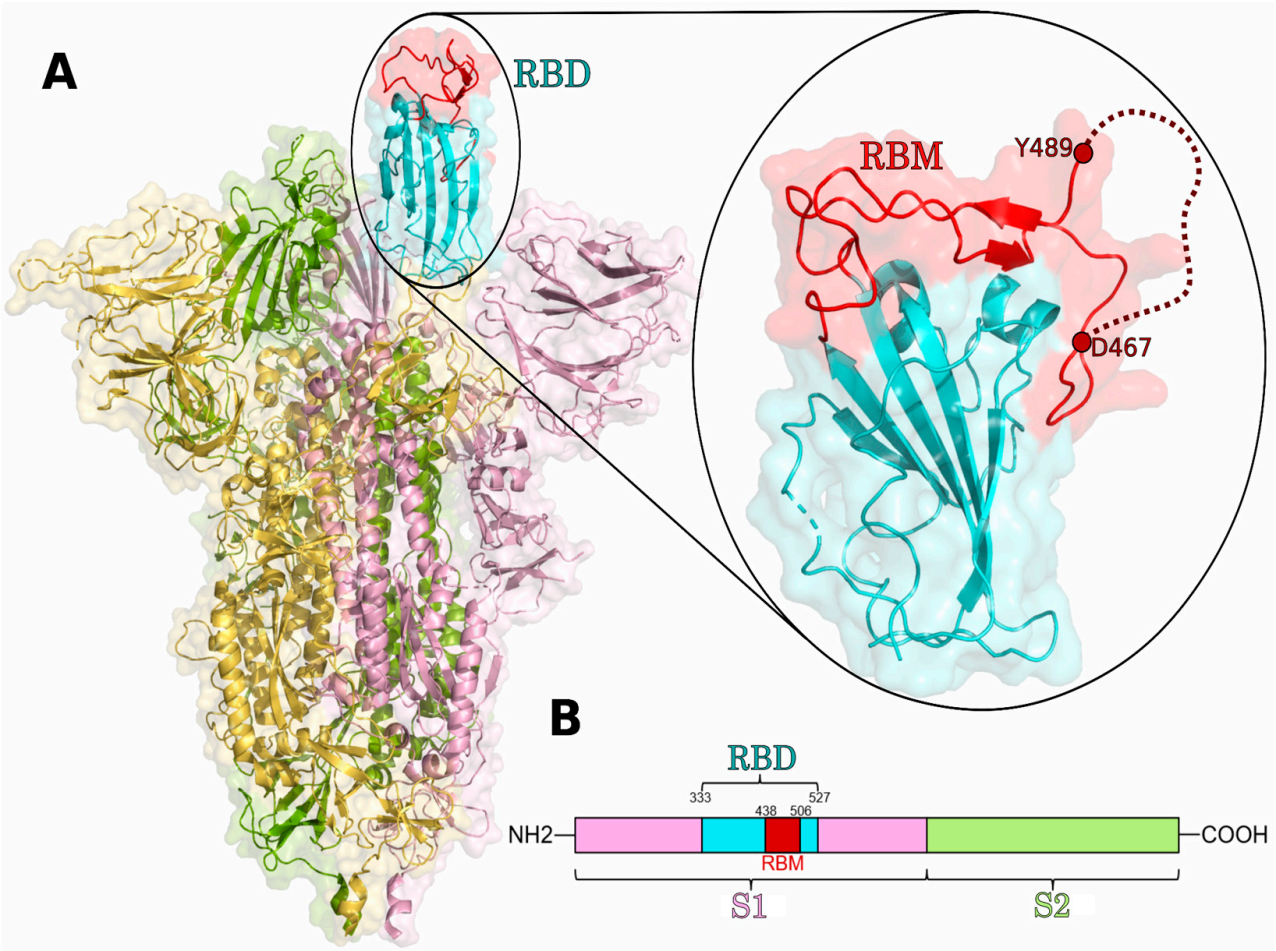
(**A**) Structure of the trimeric S-protein (each monomer is depicted with its own color) (PDB ID: 7fcd [18]). Insertion gives a closer look at the RBD (receptor-binding domain) (cyan) and its RBM (receptor binding motif) region (red); a dotted line connects amino acids D467 and Y489, indicating the commonly missing structural data. (**B**) Scheme of the domain architecture of S-protein superimposed on a primary structure.

The interaction of S-protein and ACE2 is a target for the development of preventive vaccines [8,9] and drugs for the treatment of coronavirus infection, including neutralizing antibodies [10,11,12]. Developed test systems allow the detection of antibody affinity to the S-protein [13], and due to such importance, these complexes are actively studied by structural methods [14,15]. S-protein has a receptor-binding domain (RBD) (Figure 1A) that is recognized by the target cell receptor [16,17].

Amino acids 438-506 compose the receptor binding motif (RBM) directly responsible for the ACE2 binding (Figure 1B). RBM is disordered and changes its structure to facilitate receptor binding [19]. To date, there are more than a thousand deposited structures containing RBD obtained mainly with X-Ray and Cryo-EM methods, but RBM either has a low resolution or is not resolved at all due to its disordered nature (Figure 1, dotted line). The native conformation of RBM could not be obtained by experimental methods [20]. Considering that RBM is a common target for neutralizing antibody development, such incompleteness of structural data limits the use of the theoretical methods of drug design.

All significant SARS-CoV-2 strains have at least several mutations in RBD [21]. In particular, the omicron strain (OM) carries 10 substitutions in its RBM compared to the wild-type (WT) variant [22] (Supplementary Materials Texts S1 and S2). Interestingly, five of them (K440, S446, N447, K478, and A484) are similar to those in the SARS-CoV-1 and in the bat variant of the virus [23].

OM has a higher affinity for ACE2 than the WT [24] but is significantly lower than the delta strain [25]. The ability of OM to remain unnoticed by the immune system for quite a long time (due to the lower affinity for antibodies) makes it more infectious than its predecessors [25]. These features should have their root in the structural changes. However, as mentioned before, the disordered nature of RBM makes the structure determination challenging for the experimental methods. Theoretical methods of molecular modeling are the tool to overcome these challenges.

Molecular dynamics studies of the interactions between the S-protein and ACE2 using full-atomic models are hampered by the large size of the system [26]. However, using partial protein structures or coarse-grained models could provide a reasonable compromise between the cost of calculations and the amount of information received [27,28]. The dynamics of mutant forms demonstrated that mutations A348T, N354D, D364Y, G476S, V483A, and S494D in RBD have a significant effect on the structure of S-protein, which can lead to a decrease in the ability to bind the receptor [29,30]. Mutations Y449A, N487A, Y489A, N501A, and Y505A make RBM more mobile in comparison to the wild-type variant [31]. The N501Y substitution significantly enhances the interaction of the spike protein with the receptor [32]. Molecular dynamics coupled with quantum chemistry have revealed the most significant amino acids in shaping the interaction between RBD and ACE2. From the RBD side, these are Q493, Y505, Q498, N501, T500, N487, Y449, F486, K417, Y489, F456, Y495, and L455, and from the ACE2 side—K353, K31, D30, D355, H34, D38, Q24, T27, Y83, Y41, and E35 [33]. The alpha variant RBD binds to ACE2 more strongly than the beta or wild-type RBD. In the beta variant, the K417N mutation reduces binding, E484K enhances it slightly, and N501Y significantly increases it, as in the alpha strain [33]. Structural comparison, molecular dynamics, and calculation of binding free energy together identified four key mutations (S477N, G496S, Q498R, and N501Y) that enhance ACE2 binding by the receptor-binding domain of the Omicron strain compared to the wild type [34]. Other RBD modifications also affect receptor binding, e.g., glycosylation at the S494 site increases the affinity of RBD for ACE2 [35]. All that data is incorporated in recent theoretical approaches for the new anti-COVID-19 drug development [36].

Another line of drug development research is aimed at studying the interactions between the SARS-CoV-2 S-protein and antibodies to understand the mechanism of binding and create novel immunotherapies. According to the coronavirus antibody database [37], there are more than 3000 discovered sequences of antibodies that bind RBD. Structures of more than 200 such RBD-antibody complexes are deposited to the Protein Data Bank. One of them is P4A1 which is a binding RBM and was chosen as an example for this work. Specifically, the heavy chain of P4A1 interacts with K417 and Y453 of S-protein by creating hydrogen bonds and hydrophobically with L485, F456, and Y489. In addition, the light chain forms a hydrogen bonds network between its LCDR1 and T500, N501, G502, Q498, Y449, and G496, and between its LCDR3 and Y505 and R403 [38]. Missense mutations in omicron (G496S, Q498R, N501Y, Y505H) could lead to critical changes in P4AI binding.

Thus, the position, orientation, and substitutions of amino acids in RBD (and specifically in RBM) can significantly affect the binding of certain antibodies and cellular receptors. In this work, we go beyond the pair interactions in RBD-ACE2/Antibody complexes and study the changes in the backbone conformation caused by multiple mutations in RBD.

## 2. Materials and Methods

### 2.1. P4A1 and RBD Variants Production and Purification

The DNA of the SARS-CoV-2 neutralizing antibody P4A1 corresponding to variable regions of the heavy and light chains was synthesized and cloned into expression plasmids containing the human IgG1 heavy-chain and kappa light-chain constant regions as described previously [38]. P4A1 was produced as a full-length IgG1 by transient transfection of suspension HEK293F cells using FreeStyle 293 medium and polyethylenemine (PEI) reagent [39]. The culture medium with P4A1 antibody was loaded on the HiTrap Protein G HP affinity column (GE Healthcare, Milwaukee, WI, USA), washed with 20X CV of PBS, and eluted with 0.1 M glycine buffer pH 2.5 according to the manufacturer’s guidelines. P4A1 was dialyzed against PBS pH 7.4 and refined by SEC with Superdex 200 10/300 GL (GE Healthcare, Milwaukee, WI, USA). The DNA of the codon-optimized RBD 330–528 aa fragment was synthesized in fusion with AviTag and 6His tag and cloned into the pFUSE vector via EcoRI and EcoRV restriction sites. RBD was produced by transient transfection of suspension HEK293F cells using FreeStyle 293 medium and polyethylenemine (PEI) reagent. RBD was purified by metal chelate affinity chromatography with HiTrap Chelating HP (GE Healthcare, Milwaukee, WI, USA), concentrated with an Amicon Ultra 10K device (Merck Millipore, Billerica, MA, USA), and refined by SEC with Superdex 75 10/300 GL (GE Healthcare, Milwaukee, WI, USA).

### 2.2. Binding Kinetics

SPR experiments were performed using the Biacore T200 system (GE Healthcare, Milwaukee, WI, USA). In brief, experiments were performed at 25 °C in an HBS-EP buffer. Wild-type (WT) and omicron (OM) G339D, S371L, S373P, S375F, K417N, N440K, G446S, S477N, T478K, E484A, Q493R, G496S, Q498R, N501Y, and Y505H RBDs were immobilized on the activated CM5 sensor chips. Binding kinetics was determined in single-cycle kinetics mode with various P4A1 concentrations in the 5–80 nm range. The results were analyzed using the 1:1 binding kinetics model by Biacore T200 Evaluation Software V. 1 according to the manufacturer’s guidelines.

### 2.3. RBD Protein Models

Graphical representation of the theoretical workflow provided in Supplementary Materials Figure S1.

The s-protein of the SARS-CoV-2 (Figure 1) is a 420–490 kDa protein [40]. For molecular dynamics, we isolated RBD (receptor binding domain)—the S-protein region from amino acids 333 to 527 (Figure 1, the sequence is presented in SI). Five starting models of the wild-type (WT) and of the omicron (OM) strain for molecular dynamics simulations were obtained using the AI model AlphaFold II (DeepMind, London, UK, 2021) [41] (see Supplementary Materials Figures S2 and S3). One WT model was selected from the Protein Data Bank (7cjf, chain C); that model is obtained from the structure of the complex with P4A1 neutralizing antibody directly bound to the RBM [38]. To improve side chain conformations, all starting models were refined by Rosetta [42] using the PackRotamerMover protocol with REF2015 score function [43,44]; protocol input options are given in the Supplementary Materials Text S3. Each top score solution was used for MD. For the further dihedral principal component analysis, a part of RBD called RBM (Figure 1, amino acids 438–506 for WT) was used.

### 2.4. Molecular Dynamics

Molecular dynamics was carried out at neutral pH using the GROMACS package [45] version 2020.1 and the a99SB-disp force field [46], which is used to model both structured and unstructured proteins [47]. An integration time step of 2 fs was used, and three-dimensional periodic boundary conditions were imposed. The temperature of 300 K and pressure of 1 atm was maintained in the system using the V-rescale [48] and Parrinello–Rahman [49] algorithms, respectively. A 12 Å cutoff radius was defined for the Coulombic and van der Waals interactions. Electrostatic effects were treated using the particle-mesh Ewald summation [50]. Proteins were solvated (water model TIP4P [51], and Na^+^ and Cl^−^ions were added to neutralize the system. Prior to MD simulations, the systems were subjected to energy minimization (5000 conjugate gradient steps), followed by heating from 5 to 300 K over the course of 5 ns. Protein and solvent molecules were coupled separately. For each model, 500 ns trajectories were calculated, making it a total of 5.5 μs for the RBD dynamics.

### 2.5. Dihedral Angle Principal Component Analysis (dPCA)

**Figure 2:**
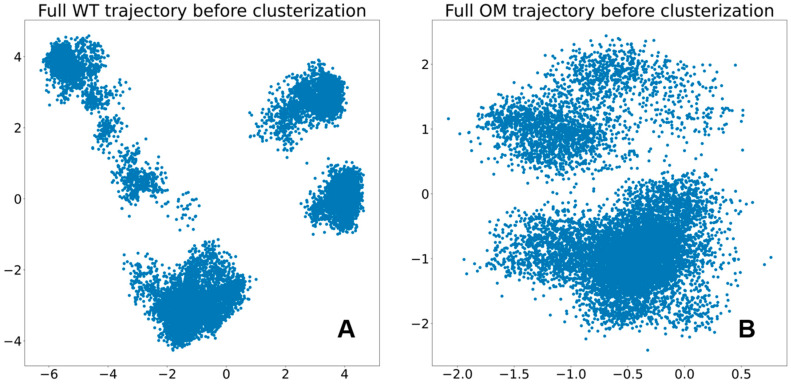
Distribution of the first two principal components of WT (**A**) and OM (**B**) RBM conformations in dPCA space.

RBM backbones of the WT (amino acids of RBD 106–172) and OM (amino acids of RBD 105–170) variants were chosen for further analysis. To equalize the initial count of dihedrals in WT and OM variants, an amino acid D134 of WT RBD was deleted. The MD trajectories were combined altogether with gmx_trjcat and processed using the dPCA algorithm [52] of the GROMACS software package to create common principal components space. After that, WT and OM frames were represented in the created space separately (Figure 2). The data obtained were subjected to K-means-based cluster analysis [53] using Python 3.9 (Supplementary Materials Figures S4 and S5).

## 3. Results and Discussion

**Figure 3:**
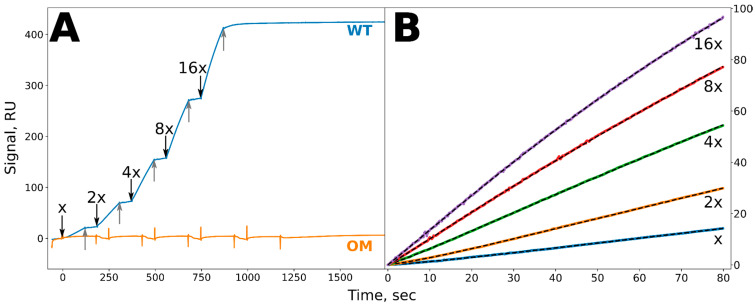
Binding kinetics between P4A1 antibody and WT or OM variants of RBD. (**A**) Binding kinetics for WT (blue) and OM (orange) RBD variants. In WT, the start of the binding phase is marked with black arrows, and the start of the dissociation phase is marked with gray arrows. Each sequential binding phase is conducted with a higher concentration of P4A1 (concentration is indicated above the corresponding arrow, x stands for 5 nm). For the OM variant, the same five-stage kinetics measurement was performed, and the start of each phase is obvious. (**B**) The binding phases are normed by the signal at the beginning. Colored curves correspond to the experimental signal, and dashed black lines represent the result of fitting with the Conformational Transition Model.

**Scheme 1:**
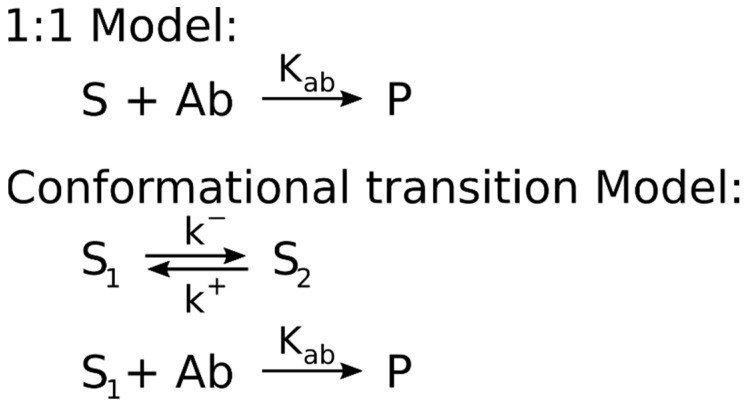
Alternative models for the antibody (Ab) binding to RBM (S). 1:1 Binding Model treats the molecular complex (P) formation process as a non-reversible second-order reaction with K_ab_ association rate constant. The Conformational Transition Model implies the presence of several antigen conformations (S1 and S2) in the ensemble of reacting particles. The transition between these is a reversible first-order reaction with k^−^ and k^+^ rate constants. Only one of the conformations (S1) can be effectively bound by the Ab in a similar to the 1:1 Model manner.

The experimental kinetics of the P4A1 binding to WT and OM variants of RBD (Figure 3A) clearly shows that this antibody is not effective in the OM case. Additionally, one can notice that the binding kinetics for WT RBD is fairly slow despite being irreversible. Fitting the experimental results with the 1:1 Langmuir Binding Model (Scheme 1) gives us a value for the K_ab_ association rate constant equal to 7.7 × 10^5^ M^−1^s^−1^. This estimate is at the lower end of the range (1 × 10^4^–1 × 10^6^ M^−1^s^−1^) known for SARS-CoV-2 neutralizing monoclonal antibodies [54]. K_ab_ depends mainly on the formation rate of the productive collision complex between antibody and antigen, and the number of these could be decreased because of the occurrence of non-productive ones. We tend to assign non-productive antibody-antigen complexes to the inner conformation. Since P4A1, with its 12 AA HCDR3, have a relatively simple structure, the divergence of conformations should be caused by several different conformations in antigen assembly. Different conformations within RBM might have different affinities for P4A1. Thus, the resulting binding kinetics might be dependent on the distribution between alternative RBM conformations.

The simplest model that satisfies the above reasoning Is shown in Scheme 1 as the “Conformational Transition Model.” It suggests the presence of two alternative RBM conformations S1 and S2, but only one (S1) can be effectively bound by the antibody (Ab). There is a dynamic balance between S1 and S2 states, and transitions correspond to k^−^ and k^+^ rate constants. The use of that model is limited due to the lack of information on the starting distribution between S1 and S2 states and uncertainty in multivariable fitting, but it could provide some qualitative insight into the proposed process. To fit the experimental data, we tried several constraints to the Conformational Transition Model: the starting point distribution [S1]:[S2] is equal to 1:5; k^−^ and k^+^ values of the same order (Figure 3B). The result gives us an estimate of 4.5 × 10^5^ M^−1^s^−1^ for a new K_ab_, which is noticeably greater than the initial K_ab_ for the 1:1 Model, and the ratio between k^−^/k^+^ and K_ab_ constants is ~50. An increase of K_ab_ suggests faster binding between the antibody and the productive conformational state of RBM, explaining a slower rate of binding by P4A1 with the appearance of alternative conformations within the antigen. The slope of the binding kinetic indicates a rapid exchange between the reactive and non-reactive conformations. 

To find hints at the existence of alternative conformations, we used the methods of molecular modeling.

**Figure 4:**
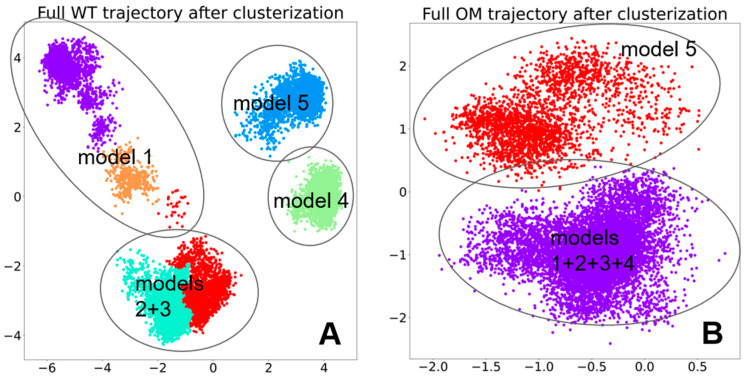
Clusterization results of the first two principal components of WT (**A**) and OM (**B**) RBM conformations in dPCA space. Clusters assigned with corresponding AlphaFold models (starting conditions).

The dPCA analysis of MD results clearly shows high conformational variation in the studied RBM assembly. The clusterization helps to categorize the structures occurring in the MD trajectories. We chose a breakdown into six clusters for the WT models and two for omicron (Figure 4). The obtained clusters are relatively stable through 500 ns dynamics, and the belonging to a particular cluster is determined by the initial model. The proximity of points in the space of principal components characterizes the similarity of the backbone structure of their RBM domains. Thus, we can assert that purple and orange clusters in Figure 4A have more similar structures than purple and green clusters.

Model 1 for WT unites two clusters (Figure 4A); cyan and red clusters are mainly a mix of models 2 and 3 and part of model 1, indicating that the circled areas of the dPCA space correspond to a relatively fast rate of conformational changes. Models 4 and 5 of WT construct two separate clusters and are not mixed with any other model. Figure 4B shows a relation between models and clusters for OM, where model 5 (red) is separated from others.

**Figure 5:**
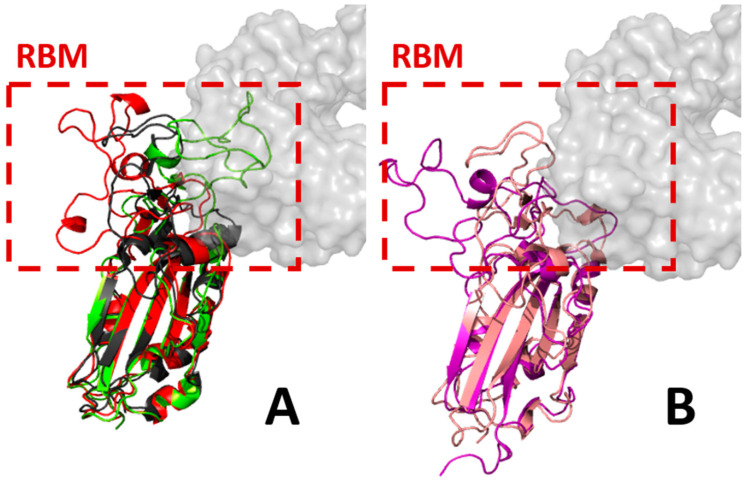
Conformations of RBD models. All models are aligned by Cα atoms of the structured part of the RBD domain (a.a. 348–468). (**A**) Wild type (model 2 is red, model 4 is green, crystal structure 7cjf is black). (**B**) OM (model 2 is purple, and model 5 is salmon). The gray transparent surface at the top right of each panel represents the P4A1 antibody aligned with 7cjf RBD.

Structures corresponding to the different clusters of WT and OM are presented in Figure 5. Multiple mutations in the omicron strain change the full RBD charge from +2 for WT to +6. However, even in models within the same strain, we can see the differences in the solution exposed surface throughout the molecular dynamics trajectory. For example, WT model 1 (purple, Figure 5A) exposes hydrophilic amino acids (e.g., G476, N486), while WT model 4 and 7cjf structures (green and black, Figure 5A) are, for most of the time, facing the contact area with hydrophobic amino acids (Y473, T478). OM model 5 (red, Figure 5B) exposes hydrophilic N475, K476, N479, and G483 and has structural similarities with WT model 1.

**Figure 6:**
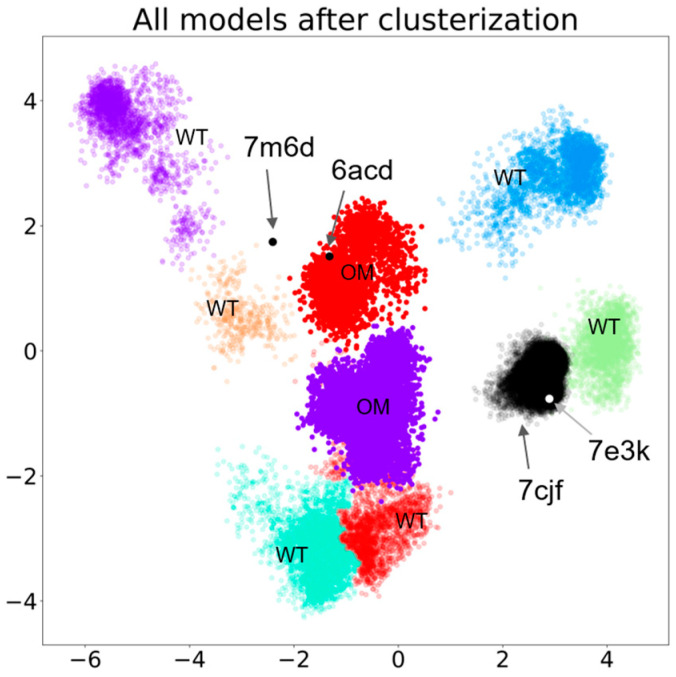
Clusterization results for omicron (bright, OM) superimposed on wild type (translucent, WT). Black translucent dots show the results of dPCA for molecular dynamics trajectory of the WT crystal structure 7cjf. Black and white bright dots show the location of crystal structures 6acd, 7e3k, and 7m6d on the map.

Notably, the dispersion of principal components is higher for WT, which is depicted in Figure 6. Comparison of the PC distribution in WT and OM variants demonstrates that the conformational space of the latter is significantly less diverse (Figure 6), showing that the RBM of OM adopts a continuum of conformations close to WT clusters of models 1, 2, and 3. It shows that despite the multiple mutations in OM, its RDB can form conformations similar to the wild-type ones.

Conducted calculations demonstrate the diversity of RBM conformations (Figure 4, Figure 5 and Figure 6), as well as an isolated character of at least four conformational states in WT and two in OM variants. The transitions between identified states are expected on a timescale greater than 500 ns.

To compare theoretical models of RBD with experimental data, we choose several crystal structures: 6acd, 7m6d, 7e3k, and 7cjf (see Supplementary Materials Table S1). In all of them, the full RBM sequence was resolved, presumably because the spatial structure of RBM was stabilized by intermolecular interactions. For 7cjf (complex of WT RBD with P4A1), RBD was separated, and 500 ns MD was performed. Notably, the RBD model from the 7cjf crystal structure exhibits the same stable (at least along 500 ns dynamics) behavior as the models obtained with AlphaFold II (DeepMind, London, UK, 2021). Although the cluster corresponding to 7cjf is close to the cluster of model 4 in WT, they did not merge, indicating the relative stability of the RBM conformation formed as a result of the interaction with the P4A1 antibody. We did not perform MD calculations for 6acd, 7m6d, and 7e3k, assuming that they will have a similar narrow distribution around the starting conformation.

Structure 6acd from Protein Data Bank is the complex of wild-type RBD with human ACE2 [55]. WT RBM in complex with ACE2 forms an “omicron-like” conformation which is indicated by the proximity of a corresponding point to the area of omicron clusters (Figure 6, 6acd marker). It could explain a higher affinity of the ACE2 receptor to the omicron strain. Structures 7m6d [56] and 7e3k [57] represent complexes of S-protein with antibodies CR3022 and 13G9, respectively. Previous studies have demonstrated that CR3022 binds wild-type RBD and has a weak affinity to omicron strain [56], while 13G9 only binds wild-type [57]. The location of these structures in Figure 6 confirms described characteristics: 7m6d is located between WT and OM clusters but closer to WT model 1, and 7e3k is close to WT model 4 and far from any omicron cluster. Furthermore, the structure 7cjf behaves like 7e3k; this fact allows us to assume that the 13G9 antibody predominantly binds WT RBM rather than OM.

## 4. Conclusions

The biochemical experiment showed that the P4A1 antibody is unable to bind the omicron variant. A low rate of RBD binding to the antibody suggests the presence of several conformations within RBM. The theoretical analysis of RBD molecular dynamics supports the suggestion of the variability of SARS-CoV-2 spike protein structures.

Obtained results may be useful in several areas. First, they could help to refine empirical structural data by providing conformational states which are superposed in the actual samples. Better experimental structures, in turn, will help further theoretical studies of interactions in RBD-antibody or RBD-ACE2 complexes. Second, obtained individual states could be used as alternative target models in a theoretical target-based drug design. Third, they show conformational changes associated with mutations in the OM variant and could be easily extended to any other variant. One can expect that the affinity to a certain neutralizing antibody should depend on the RBM state in the collision complex. Depending on the affinity distribution, the rate of conformational transitions in RBD assembly could have a heavy impact on the observable dynamics of antibody binding. Thus, based on the short distance in the dPCA space in the case of the P4A1 antibody, the state corresponding to the WT model 4 (Figure 6, translucent green) should have a higher affinity. Comparing similar distances to OM models gives a clear idea of why the P4A1 antibody is non-effective with that variant. Those predictions can facilitate further fundamental studies of antibodies and the creation of novel monoclonal antibodies-based immunotherapies. The best-case scenario is the development of an antibody that will bind to any variants of the virus and do so in a fast and irreversible manner. Any new variant should preserve its ability to be bound to ACE2, which imposes significant structural constraints on RBM. That makes RBM a very promising universal target for drug development. The disordered nature of that region imposes significant complications for the theoretical approaches. The approach proposed in this paper aims to overcome these difficulties.

## Data Availability

3D-structures for obtained clusters centroids are available upon request via email.

## Supplementary Materials

The following supporting information can be downloaded at: https://www.mdpi.com/article/10.3390/biomedicines10123233/s1, Figure S1: Representation of the computational workflow; Figure S2: Sequence coverage for the modeling of WT (A) and OM (B) variants of RBD using Alpha Fold II; Figure S3: pLDDT score for five top ranking models obtained with Alpha Fold II for WT (A) and OM (B) variants of RBD; Figure S4: Results of clusterization of the WT trajectory in the basis of principal components. Cut-off line is added; Figure S5: Results of clusterization of the omicron trajectory in the basis of principal components. Cut-off line is added; Table S1: Description of RBD structures from Protein Data Bank; Text S1: RBD wild type sequence; Text S2: RBD omicron sequence; Text S3: Rosetta repack protocol options.

## References

[1] Khalifa S.A.M., Swilam M.M., El-Wahed A.A.A., Du M., El-Seedi H.H.R., Kai G., Masry S.H.D., Abdel-Daim M.M., Zou X., Halabi M.F. (2021). Beyond the Pandemic: COVID-19 Pandemic Changed the Face of Life. Int. J. Environ. Res. Public Health.

[2] Khalifa S.A.M., Mohamed B.S., Elashal M.H., Du M., Guo Z., Zhao C., Musharraf S.G., Boskabady M.H., El-Seedi H.H.R., Efferth T. (2020). Comprehensive Overview on Multiple Strategies Fighting COVID-19. Int. J. Environ. Res. Public Health.

[3] Ershov A.V., Surova V.D., Dolgikh V.T., Dolgikh T.I. (2021). Cytokine Storm in the Novel Coronavirus Infection and Methods of Its Correction. Antibiot Chemother.

[4] Cheng H., Wang Y., Wang G. (2020). Organ-protective Effect of Angiotensin-converting Enzyme 2 and Its Effect on the Prognosis of COVID-19. J. Med. Virol..

[5] Hanff T.C., Harhay M.O., Brown T.S., Cohen J.B., Mohareb A.M. (2020). Is There an Association Between COVID-19 Mortality and the Renin-Angiotensin System? A Call for Epidemiologic Investigations. Clin. Infect. Dis..

[6] Colunga Biancatelli R.M.L., Solopov P.A., Sharlow E.R., Lazo J.S., Marik P.E., Catravas J.D. (2021). The SARS-CoV-2 Spike Protein Subunit S1 Induces COVID-19-like Acute Lung Injury in Κ18-HACE2 Transgenic Mice and Barrier Dysfunction in Human Endothelial Cells. Am. J. Physiol.-Lung Cell. Mol. Physiol..

[7] Gattinoni L., Gattarello S., Steinberg I., Busana M., Palermo P., Lazzari S., Romitti F., Quintel M., Meissner K., Marini J.J. (2021). COVID-19 Pneumonia: Pathophysiology and Management. Eur. Respir. Rev..

[8] Kleanthous H., Silverman J.M., Makar K.W., Yoon I.-K., Jackson N., Vaughn D.W. (2021). Scientific Rationale for Developing Potent RBD-Based Vaccines Targeting COVID-19. NPJ Vaccines.

[9] Chen W.-H., Hotez P.J., Bottazzi M.E. (2020). Potential for Developing a SARS-CoV Receptor-Binding Domain (RBD) Recombinant Protein as a Heterologous Human Vaccine against Coronavirus Infectious Disease (COVID)-19. Hum. Vaccin Immunother.

[10] Prajapat M., Shekhar N., Sarma P., Avti P., Singh S., Kaur H., Bhattacharyya A., Kumar S., Sharma S., Prakash A. (2020). Virtual Screening and Molecular Dynamics Study of Approved Drugs as Inhibitors of Spike Protein S1 Domain and ACE2 Interaction in SARS-CoV-2. J. Mol. Graph. Model..

[11] Bertoglio F., Fühner V., Ruschig M., Heine P.A., Abassi L., Klünemann T., Rand U., Meier D., Langreder N., Steinke S. (2021). A SARS-CoV-2 Neutralizing Antibody Selected from COVID-19 Patients Binds to the ACE2-RBD Interface and Is Tolerant to Most Known RBD Mutations. Cell Rep..

[12] Lv Z., Deng Y.-Q., Ye Q., Cao L., Sun C.-Y., Fan C., Huang W., Sun S., Sun Y., Zhu L. (2020). Structural Basis for Neutralization of SARS-CoV-2 and SARS-CoV by a Potent Therapeutic Antibody. Science.

[13] Kyosei Y., Namba M., Yamura S., Takeuchi R., Aoki N., Nakaishi K., Watabe S., Ito E. (2020). Proposal of De Novo Antigen Test for COVID-19: Ultrasensitive Detection of Spike Proteins of SARS-CoV-2. Diagnostics.

[14] Benton D.J., Wrobel A.G., Roustan C., Borg A., Xu P., Martin S.R., Rosenthal P.B., Skehel J.J., Gamblin S.J. (2021). The Effect of the D614G Substitution on the Structure of the Spike Glycoprotein of SARS-CoV-2. Proc. Natl. Acad. Sci. USA.

[15] Wrobel A.G., Benton D.J., Roustan C., Borg A., Hussain S., Martin S.R., Rosenthal P.B., Skehel J.J., Gamblin S.J. (2022). Evolution of the SARS-CoV-2 Spike Protein in the Human Host. Nat. Commun..

[16] Verma J., Subbarao N. (2021). A Comparative Study of Human Betacoronavirus Spike Proteins: Structure, Function and Therapeutics. Arch. Virol..

[17] Liu K., Tan S., Niu S., Wang J., Wu L., Sun H., Zhang Y., Pan X., Qu X., Du P. (2021). Cross-Species Recognition of SARS-CoV-2 to Bat ACE2. Proc. Natl. Acad. Sci. USA.

[18] Zhang S., Liang Q., He X., Zhao C., Ren W., Yang Z., Wang Z., Ding Q., Deng H., Wang T. (2022). Loss of Spike N370 Glycosylation as an Important Evolutionary Event for the Enhanced Infectivity of SARS-CoV-2. Cell Res..

[19] Choi K.-E., Kim J.-M., Rhee J., Park A.K., Kim E.-J., Kang N.S. (2021). Molecular Dynamics Studies on the Structural Characteristics for the Stability Prediction of SARS-CoV-2. Int. J. Mol. Sci..

[20] Olia A.S., Tsybovsky Y., Chen S.J., Liu C., Nazzari A.F., Ou L., Wang L., Kong W.-P., Leung K., Liu T. (2021). SARS-CoV-2 S2P Spike Ages through Distinct States with Altered Immunogenicity. J. Biol. Chem..

[21] Harvey W.T., Carabelli A.M., Jackson B., Gupta R.K., Thomson E.C., Harrison E.M., Ludden C., Reeve R., Rambaut A., Peacock S.J. (2021). SARS-CoV-2 Variants, Spike Mutations and Immune Escape. Nat. Rev. Microbiol..

[22] Tian D., Sun Y., Xu H., Ye Q. (2022). The Emergence and Epidemic Characteristics of the Highly Mutated SARS-CoV-2 Omicron Variant. J. Med. Virol..

[23] Islam F., Dhawan M., Nafady M.H., Emran T.B., Mitra S., Choudhary O.P., Akter A. (2022). Understanding the Omicron Variant (B.1.1.529) of SARS-CoV-2: Mutational Impacts, Concerns, and the Possible Solutions. Ann. Med. Surg..

[24] Nguyen H.L., Thai N.Q., Nguyen P.H., Li M.S. (2022). SARS-CoV-2 Omicron Variant Binds to Human Cells More Strongly than the Wild Type: Evidence from Molecular Dynamics Simulation. J. Phys. Chem. B.

[25] Wu L., Zhou L., Mo M., Liu T., Wu C., Gong C., Lu K., Gong L., Zhu W., Xu Z. (2022). SARS-CoV-2 Omicron RBD Shows Weaker Binding Affinity than the Currently Dominant Delta Variant to Human ACE2. Signal Transduct Target. Ther..

[26] Padhi A.K., Rath S.L., Tripathi T. (2021). Accelerating COVID-19 Research Using Molecular Dynamics Simulation. J. Phys. Chem. B.

[27] Ma B., Zhang Z., Li Y., Lin X., Gu N. (2022). Evaluation of Interactions between SARS-CoV-2 RBD and Full-Length ACE2 with Coarse-Grained Molecular Dynamics Simulations. J. Chem Inf. Model..

[28] Leong T., Voleti C., Peng Z. (2021). Coarse-Grained Modeling of Coronavirus Spike Proteins and ACE2 Receptors. Front. Phys..

[29] Ahamad S., Hema K., Gupta D. (2021). Structural Stability Predictions and Molecular Dynamics Simulations of RBD and HR1 Mutations Associated with SARS-CoV-2 Spike Glycoprotein. J. Biomol. Struct. Dyn..

[30] Yang Y., Zhang Y., Qu Y., Zhang C., Liu X.-W., Zhao M., Mu Y., Li W. (2021). Key Residues of the Receptor Binding Domain in the Spike Protein of SARS-CoV-2 Mediating the Interactions with ACE2: A Molecular Dynamics Study. Nanoscale.

[31] Dehury B., Raina V., Misra N., Suar M. (2021). Effect of Mutation on Structure, Function and Dynamics of Receptor Binding Domain of Human SARS-CoV-2 with Host Cell Receptor ACE2: A Molecular Dynamics Simulations Study. J. Biomol. Struct Dyn..

[32] Tian F., Tong B., Sun L., Shi S., Zheng B., Wang Z., Dong X., Zheng P. (2021). N501Y Mutation of Spike Protein in SARS-CoV-2 Strengthens Its Binding to Receptor ACE2. Elife.

[33] Jawad B., Adhikari P., Podgornik R., Ching W.-Y. (2021). Key Interacting Residues between RBD of SARS-CoV-2 and ACE2 Receptor: Combination of Molecular Dynamics Simulation and Density Functional Calculation. J. Chem. Inf. Model..

[34] Rath S.L., Padhi A.K., Mandal N. (2022). Scanning the RBD-ACE2 Molecular Interactions in Omicron Variant. Biochem. Biophys. Res. Commun..

[35] Rahnama S., Azimzadeh Irani M., Amininasab M., Ejtehadi M.R. (2021). S494 O-Glycosylation Site on the SARS-CoV-2 RBD Affects the Virus Affinity to ACE2 and Its Infectivity; a Molecular Dynamics Study. Sci. Rep..

[36] Ford C.T., Jacob Machado D., Janies D.A. (2022). Predictions of the SARS-CoV-2 Omicron Variant (B.1.1.529) Spike Protein Receptor-Binding Domain Structure and Neutralizing Antibody Interactions. Front. Virol..

[37] Raybould M.I.J., Kovaltsuk A., Marks C., Deane C.M. (2021). CoV-AbDab: The Coronavirus Antibody Database. Bioinformatics.

[38] Guo Y., Huang L., Zhang G., Yao Y., Zhou H., Shen S., Shen B., Li B., Li X., Zhang Q. (2021). A SARS-CoV-2 Neutralizing Antibody with Extensive Spike Binding Coverage and Modified for Optimal Therapeutic Outcomes. Nat. Commun..

[39] Portolano N., Watson P.J., Fairall L., Millard C.J., Milano C.P., Song Y., Cowley S.M., Schwabe J.W.R. (2014). Recombinant Protein Expression for Structural Biology in HEK 293F Suspension Cells: A Novel and Accessible Approach. J. Vis. Exp..

[40] Herrera N.G., Morano N.C., Celikgil A., Georgiev G.I., Malonis R.J., Lee J.H., Tong K., Vergnolle O., Massimi A.B., Yen L.Y. (2021). Characterization of the SARS-CoV-2 S Protein: Biophysical, Biochemical, Structural, and Antigenic Analysis. ACS Omega.

[41] Jumper J., Evans R., Pritzel A., Green T., Figurnov M., Ronneberger O., Tunyasuvunakool K., Bates R., Žídek A., Potapenko A. (2021). Highly Accurate Protein Structure Prediction with AlphaFold. Nature.

[42] Leman J.K., Weitzner B.D., Lewis S.M., Adolf-Bryfogle J., Alam N., Alford R.F., Aprahamian M., Baker D., Barlow K.A., Barth P. (2020). Macromolecular Modeling and Design in Rosetta: Recent Methods and Frameworks. Nat. Methods.

[43] Park H., Bradley P., Greisen P., Liu Y., Mulligan V.K., Kim D.E., Baker D., DiMaio F. (2016). Simultaneous Optimization of Biomolecular Energy Functions on Features from Small Molecules and Macromolecules. J. Chem. Theory Comput..

[44] Alford R.F., Leaver-Fay A., Jeliazkov J.R., O’Meara M.J., DiMaio F.P., Park H., Shapovalov M.V., Renfrew P.D., Mulligan V.K., Kappel K. (2017). The Rosetta All-Atom Energy Function for Macromolecular Modeling and Design. J. Chem. Theory Comput..

[45] Abraham M.J., Murtola T., Schulz R., Páll S., Smith J.C., Hess B., Lindah E. (2015). Gromacs: High Performance Molecular Simulations through Multi-Level Parallelism from Laptops to Supercomputers. SoftwareX.

[46] Robustelli P., Piana S., Shaw D.E. (2018). Developing a Molecular Dynamics Force Field for Both Folded and Disordered Protein States. Proc. Natl. Acad. Sci. USA.

[47] Mamchur A.A., Moiseenko A.V., Panina I.S., Yaroshevich I.A., Kudryavtseva S.S., Pichkur E.B., Sokolova O.S., Muronetz V.I., Stanishneva-Konovalova T.B. (2021). Structural and Computational Study of the Groel–Prion Protein Complex. Biomedicines.

[48] Bussi G., Donadio D., Parrinello M. (2007). Canonical Sampling through Velocity Rescaling. J. Chem. Phys..

[49] Parrinello M., Rahman A. (1981). Polymorphic Transitions in Single Crystals: A New Molecular Dynamics Method. J. Appl. Phys..

[50] Essman U., Perera L., Berkowitz T., Darden T., Lee H., Pedersen L.G. (1995). A Smooth Particle Mesh Ewald Method. J. Chem. Phys..

[51] Jorgensen W.L., Chandrasekhar J., Madura J.D., Impey R.W., Klein M.L. (1983). Comparison of Simple Potential Functions for Simulating Liquid Water. J. Chem. Phys..

[52] Mu Y., Nguyen P.H., Stock G. (2004). Energy Landscape of a Small Peptide Revealed by Dihedral Angle Principal Component Analysis. Proteins Struct. Funct. Bioinform..

[53] Hartigan J.A., Wong M.A. (1979). Algorithm AS 136: A K-Means Clustering Algorithm. Appl. Stat..

[54] Jones B.E., Brown-Augsburger P.L., Corbett K.S., Westendorf K., Davies J., Cujec T.P., Wiethoff C.M., Blackbourne J.L., Heinz B.A., Foster D. (2021). The Neutralizing Antibody, LY-CoV555, Protects against SARS-CoV-2 Infection in Nonhuman Primates. Sci. Transl. Med..

[55] Song W., Gui M., Wang X., Xiang Y. (2018). Cryo-EM Structure of the SARS Coronavirus Spike Glycoprotein in Complex with Its Host Cell Receptor ACE2. PLoS Pathog..

[56] Scheid J.F., Barnes C.O., Eraslan B., Hudak A., Keeffe J.R., Cosimi L.A., Brown E.M., Muecksch F., Weisblum Y., Zhang S. (2021). B Cell Genomics behind Cross-Neutralization of SARS-CoV-2 Variants and SARS-CoV. Cell.

[57] Li T., Han X., Gu C., Guo H., Zhang H., Wang Y., Hu C., Wang K., Liu F., Luo F. (2021). Potent SARS-CoV-2 Neutralizing Antibodies with Protective Efficacy against Newly Emerged Mutational Variants. Nat. Commun..

