# Supplementary material for: Conformational Dynamics of the Receptor-Binding Domain of the SARS-CoV-2 Spike Protein
Source: Biomedicines. 2022 Dec 12;10(12):3233. doi: 10.3390/biomedicines10123233 (PMC9775641; doi:10.3390/biomedicines10123233)
Supplement: Supplementary file 1 [file biomedicines-10-03233-s001.zip › biomedicines-1892053-supplementary.pdf]

Conformational dynamics of the receptor-binding domain of the SARS-CoV-2 spike protein

Aleksandra A. Mamchur [1], Tatiana B. Stanishneva-Konovalova 2 , Yuliana A. Mokrushina [3], Viktoria A. Abrikosova [3], Yu Guo [4], Hongkai Zhang [4], Stanislav S. Terekhov [3], Ivan V. Smirnov [3] and Igor A. Yaroshevich [1,3,*]

1 Department of biophysics, Faculty of Biology, Lomonosov Moscow State University, Moscow 119991, Russia
2 Department of bioengineering, Faculty of Biology, Lomonosov Moscow State University, Moscow 119991, Russia
3 Shemyakin-Ovchinnikov Institute of Bioorganic Chemistry, Russian Academy of Sciences, Moscow 117997, Russia
4 State Key Laboratory of Medicinal Chemical Biology, College of Pharmacy, Nankai University, Tianjin 300071, China
* Correspondence: iyapromo@gmail.com

Supplementary information

Computational workflow details

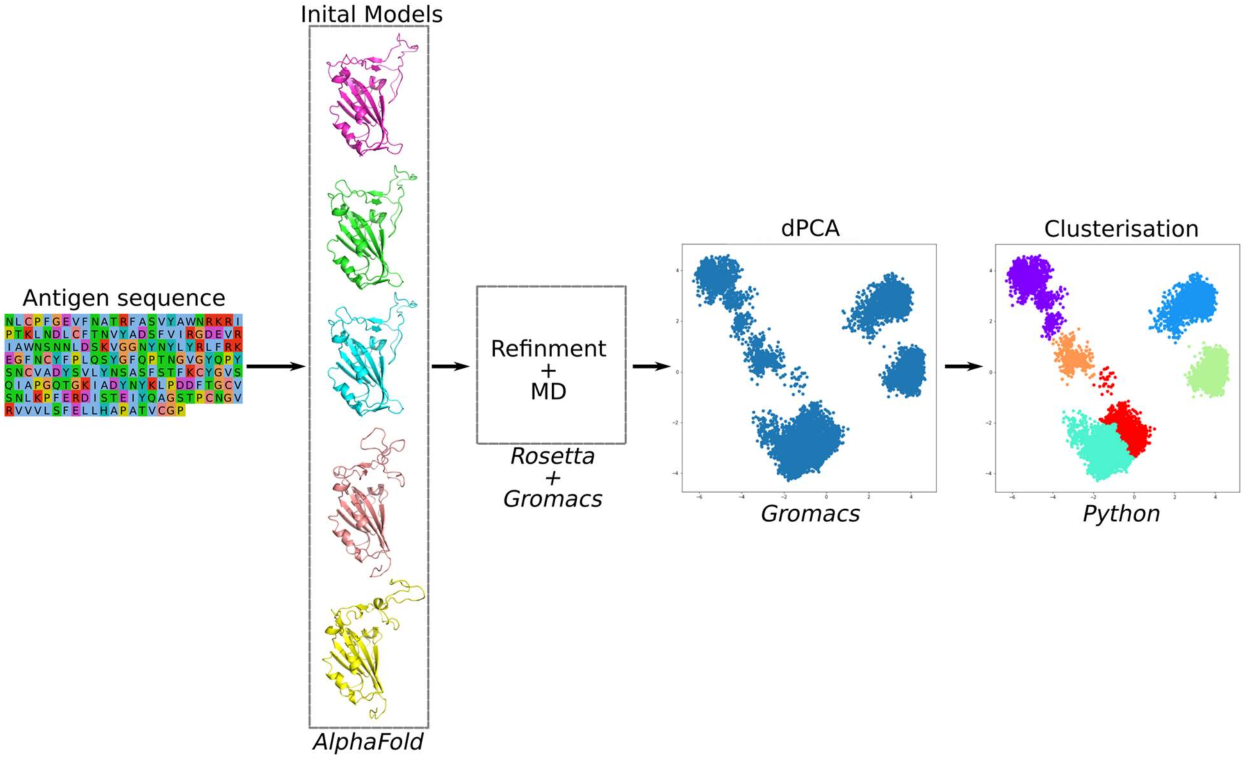

SI Figure S1. Representation of the computational workflow

Used amino acid sequences

Text S1. WT RBD amino acid sequence

LCPFGEVFNATRFASVYAWNRKRISNCVADYSVLYNSASFSTFKCYGVSPTKLNDLCFTNVYADSFV IRGDEVRQIAPGQTGKIADYNYKLPDDFTGCVIAWNSNNLDSKVGGNYNYLYRLFRKSNLKPFER DISTEIYQAGSTPCNGVEGFNCYFPLQSYGFQPTNGVGYQPYRVVVLSFELLHAPATVCGP

Text S2. Omicron RBD amino acid sequence

LCPFDEVFNATRFASVYAWNRKRISNCVADYSVLYNLAPFFTFKCYGVSPTKLNDLCFTNVYADSF VIRGDEVRQIAPGQTGNIADYNYKLPDDFTGCVIAWNSNKLDSKVSGNYNYLYRLFRKSNLKPFE RISTEIYQAGNKPCNGVAGFNCYFPLRSYSFRPTYGVGHQPYRVVVLSFELLHAPATVCGP

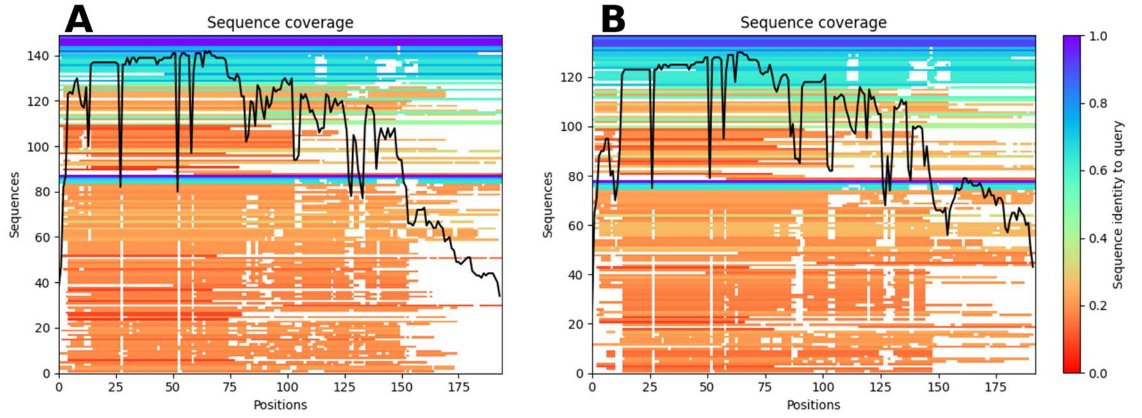

SI Figure S2. Sequence coverage for the modeling of WT (A) and OM (B) variants of RBD using Alpha Fold II.

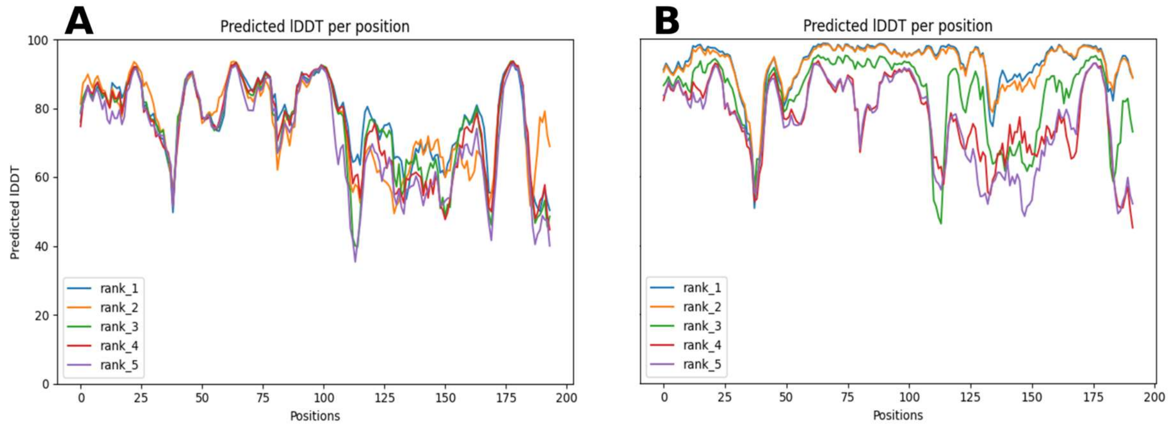

SI Figure S3. pLDDT score for five top ranking models obtained with Alpha Fold II for WT (A) and OM (B) variants of RBD.

Rosetta repack protocol details

Text S3. Rosetta repack protocol options

```
<ROSETTASCRIPTS>
	<SCOREFXNS>
	</SCOREFXNS>
	<TASKOPERATIONS>
		<InitializeFromCommandline name="ifcl" />
		<RestrictToRepacking name="rtr" />
	</TASKOPERATIONS>
	<FILTERS>
	</FILTERS>
	<MOVERS>
		<
		PackRotamersMover name="repack"
		scorefxn="REF2015" task_operations="ifcl,rtr"
		/>
		<
		MinMover name="minimize_sc"
		scorefxn="REF2015"
		chi="1" bb="0"
		jump="0"
		type="dfpmin_armijo_nonmonotone"
		tolerance="0.0001"
```

```
                    />
            </MOVERS>
            <APPLY_TO_POSE>
            </APPLY_TO_POSE>
            <PROTOCOLS>
                    <Add mover="repack" />
                    <Add mover="minimize_sc" />
            </PROTOCOLS>
    </ROSETTASCRIPTS>
```

#Rosetta repack options#:

```
-in:file:fullatom
-out:file:fullatom
-linmem_ig 10
-ex1
-ex2
-use_input_sc
-score:weights ref2015.wts
```

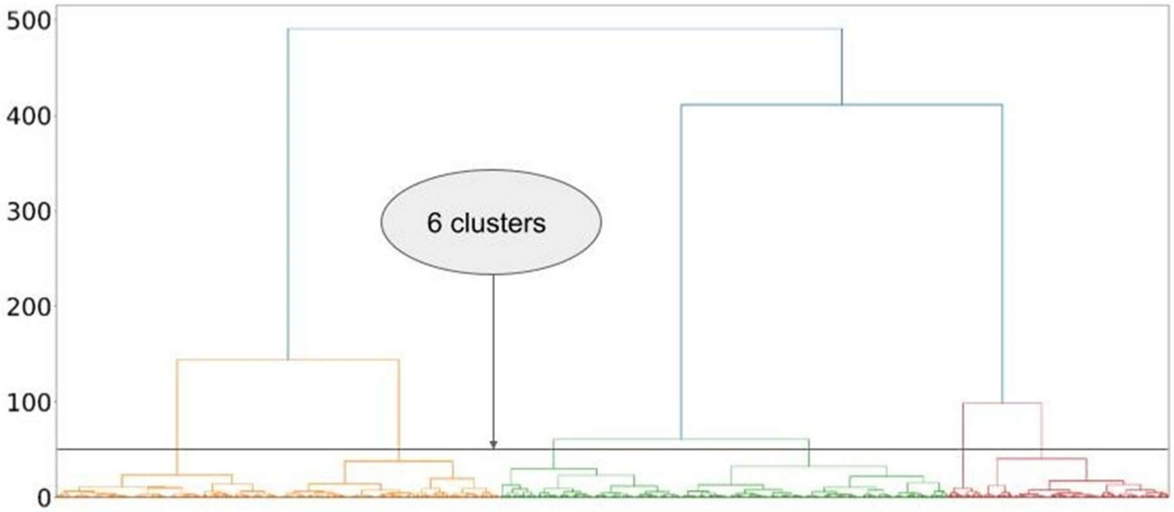

SI Figure S4. Results of clusterization of the WT trajectory in the basis of principal components. Cut-off line is added.

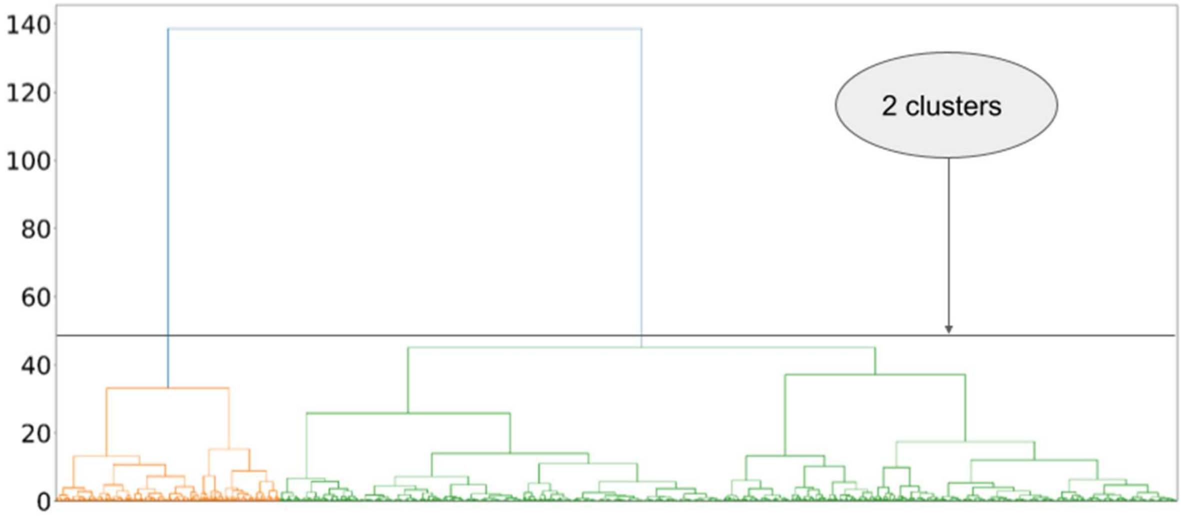

SI Figure S5. Results of clusterization of the omicron trajectory in the basis of principal components. Cut-off line is added.

SI Table S1. Description of RBD structures from Protein Data Bank

**PDB code**	**Description**	**Method**	**Resolution**
6acd	SARS-CoV spike glycoprotein in complex with ACE2	Cryo-EM	3.90 Å
7cjk	SARS-CoV-2 RBD in complex with a neutralizing antibody P4A1	X-RAY DIFFRACTION	2.11 Å
7e3k	SARS-CoV-2 RBD in complex with a neutralizing antibody 13G9	Cryo-EM	3.90 Å
7m6d	SARS-CoV-2 RBD in complex with neutralizing antibodies CR3022	X-RAY DIFFRACTION	3.10 Å

3D-structures for obtained clusters centroids are available upon request.
